# Blinatumomab-induced macrophage activating syndrome (MAS) in adult with B-cell acute lymphoblastic leukemia (B-ALL)

**DOI:** 10.1007/s00277-024-05795-9

**Published:** 2024-05-24

**Authors:** Adam Braun, Salman Otoukesh, Jose Tinajero, Guido Marcucci, Ibrahim Aldoss

**Affiliations:** Hematology and HCT, City of Hope Comprehensive Cancer Center, Duarte, USA

**Keywords:** Cellular therapies, Acute lymphoblastic leukemia, Macrophage activation syndrome, Blinatumomab

## Abstract

Blinatumomab as a single agent has demonstrated superiority over salvage chemotherapy in patients with relapsed and refractory B-cell acute lymphoblastic leukemia (B-ALL), with manageable safety and efficacy. Though known to have anticipated drug toxicities including cytokine release syndrome (CRS) and neurotoxicity, there is only one prior report of macrophage activating syndrome (MAS) due to blinatumomab. Case Presentation: We report the first case of blinatumomab-induced MAS in an adult. The patient presented with fever, cough, and weakness on the second cycle of blinatumomab. Complete blood count was notable for severe leukopenia, with comprehensive metabolic panel notable for elevated alkaline phosphatase, AST, ALT, LDH, and hyperferritinemia consistent with MAS. The patient was already in MRD-negative remission at presentation with MAS. She responded rapidly to withholding the drug and administration of both tocilizumab and dexamethasone. She was able to restart therapy with blinatumomab dosed at 9 mcg/day with no recurrence of symptoms. Though MAS is not an expected association with blinatumomab, the risk for CRS is. Secondary MAS in this case likely shares a mechanism with other hyperinflammatory conditions. Management includes holding the offending agent, like blinatumomab, and administering tocilizumab and dexamethasone. Future research will be needed to predict which patients are at highest risk to develop MAS after similar T-cell therapies.

## Introduction

The management of B-cell acute lymphoblastic leukemia (B-ALL) has evolved recently with the introduction of novel monoclonal antibody and immune-based therapies in relapsed/refractory (r/r) disease [1, 2]. Blinatumomab as a single agent has demonstrated superiority over salvage chemotherapy in patients with r/r B-ALL [1] and led to high rate of minimal residual disease (MRD) in adults with r/r MRD-positive ALL in the BLAST study [3]. Blinatumomab treatment has anticipated toxicities including cytokine release syndrome (CRS) and neurotoxicity. Only one child has been reported with MAS while receiving blinatumomab therapy, though it is a possible adverse event per the Summary of Product Characteristics at a rate > = 1/1000-<1/100 [4]. Here, we report a second case of blinatumomab-induced MAS in an adult patient with Ph + ALL treated for persistent MRD disease.

## Case presentation

This is a 59-year-old woman who presented with leukocytosis, with initial white blood cell count of 390 K/uL and 77% circulating blasts. Bone marrow biopsy showed hypercellular marrow (95%) with 80% involvement by B-cell ALL. Flow cytometry demonstrated lymphoblasts expressing CD19. Molecular analysis showed BCR::ABL1 p210 fusion, IKZF1 loss, ABL1 gene A350D mutation, and RUNX1 p.G165R mutation. She underwent induction per PhALLCON protocol [5]. Day 21 bone marrow biopsy showed MRD-positive by PCR for BCR::ABL1 at 0.77%. She was started on blinatumomab in combination with ponatinib [6]. During the first cycle, she tolerated standard 9 mcg/day dosing titrated up to 28 mcg/day. MRD assessment at cycle 1 day 38 was negative.

The second cycle of blinatumomab started at 28 mcg/day. She had no evidence of cytopenia, with normal liver and kidney function. On day 5 of cycle 2, she was ill-appearing with body aches, 101.7 F, cough and weakness. Her complete blood count was notable for white blood cell count of 0.52 K/uL. Comprehensive metabolic panel was notable for elevated alkaline phosphatase 169 U/L (36–126), ALT 62 U/L (7–56), AST 110 U/L (15–46) and lactate dehydrogenase 853 U/L (140–271). Laboratory values for alkaline phosphatase, ALT, AST, and LDH continued to increase. Ferritin was reported as > 22,500 ng/mL (10–290), along with C reactive protein 148 (< 5), and triglyceride peak was 245 mg/dL (< 150). Fibrinogen nadir was 273 mg/dL (140–410). Given suspicion of MAS, soluble IL-2R was found to be 1832.3 pg/mL (175.3-858.2). Infectious workup including viral serologic testing for EBV, CMV, HIV was negative. At this time, the patient had criteria for macrophage activation syndrome (MAS).

**Fig. 1:**
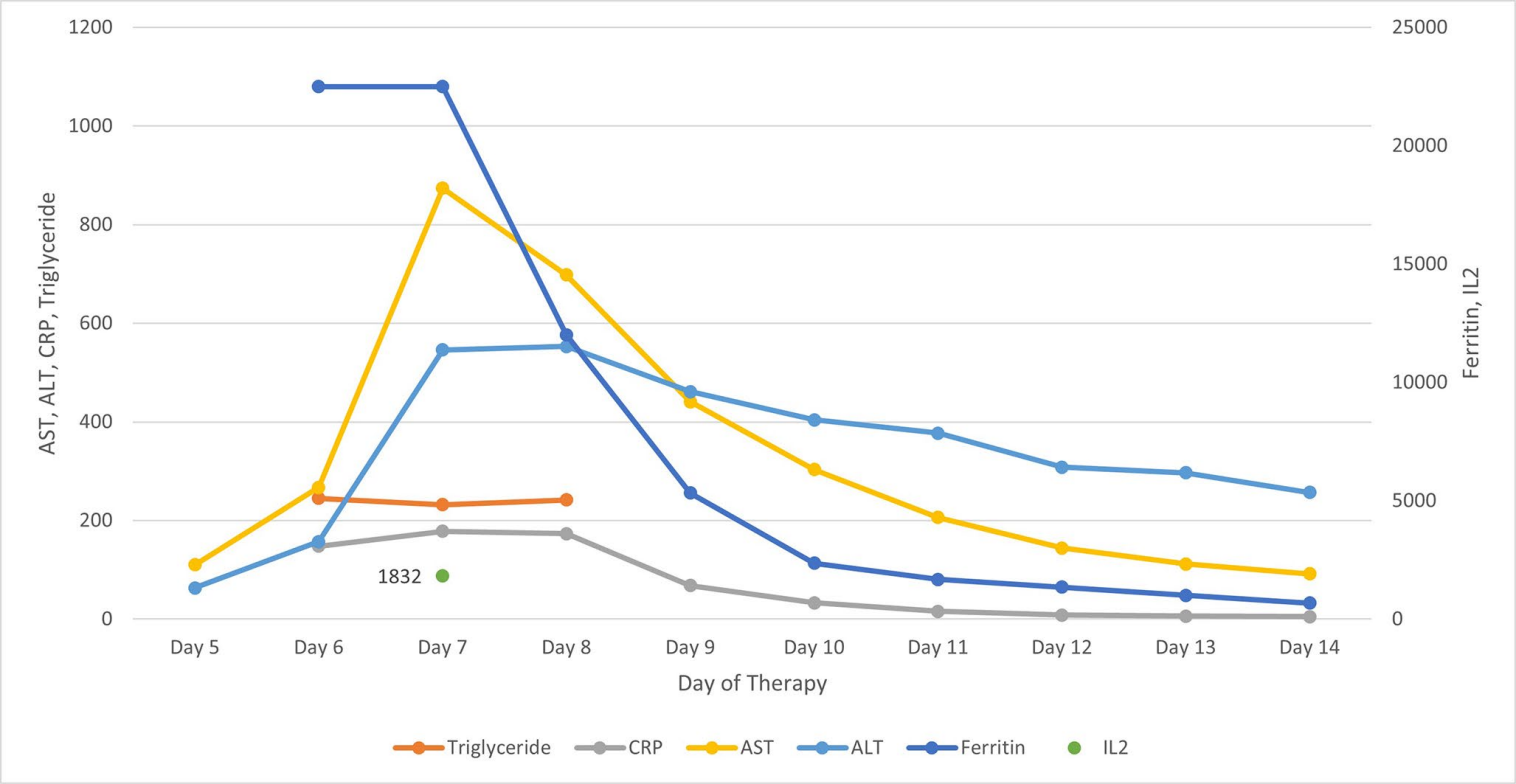
Laboratory values by day of blinatumomab therapy in cycle 2. All markers of inflammation due to MAS improved after a single intravenous dose each of tocilizumab 8 mg/kg and dexamethasone 10 mg

Blinatumomab and ponatinib were held on Cycle 2, Day 9. She was treated with one dose each of tocilizumab 8 mg/kg and dexamethasone 10 mg intravenously on day 10. Laboratory values resolved by day 10 (shown in Fig. 1). After holding for 6 days, Blinatumomab infusion was restarted at 9 mcg for 18 days with ponatinib 30 mg daily to complete cycle 2. The end of cycle 2 bone marrow biopsy showed complete molecular remission.

## Discussion

Secondary hemophagocytic lymphiohistiocytosis / macrophage activating syndrome (HLH/MAS) is an immune-mediated cytokine storm that has been increasingly reported as a toxicity of immunotherapy, more frequently encountered with cellular therapy in r/r hematological malignancies, but rarely been reported following blinatumomab. Fevers, hyperferritinemia, hypertriglyceridemia, coagulopathy, organomegaly and cytopenias are the common signs of MAS. One tool is the HScore to assist with diagnosis, with sensitivity and specificity above 90% at a score greater than 169 [7, 8]. In this case, the HScore was calculated as 194. Pathogenesis of secondary HLH/MAS after T cell therapies is like other hyperinflammatory conditions. HLH/MAS incidence after CAR-T is estimated at 1% overall, but there are minimal studies of this outcome in ALL [3]. One surprising feature of this case was the onset of MAS occurred while MRD negative, and not with measurable disease, which differed from the prior case reported. This may suggest the limitation of MRD assessment in predicting hyperinflammatory response to blinatumomab or other T-cell therapies. Currently, there is not a validated risk assessment for development of HLH/MAS, but there are successful management strategies.

Treatment of HLH/MAS in this case combined holding blinatumomab and administering a single dose of tocilizumab with dexamethasone. Contrasted with CAR T-cells, blinatumomab has a short half-life, and holding it may shut down the inflammatory process feeding MAS. Recent data suggests that tocilizumab does not significantly diminish the anticancer properties of T cell redirection therapy [9]. Tocilizumab should not be avoided for concerns about losing anticancer efficacy. Once recognized, HLH/MAS is treatable and should begin as soon as there is high index of suspicion, as mortality rates can be as high as 40% [10]. Fortunately, she responded to HLH/MAS therapy with complete resolution of symptoms and laboratory markers of disease.

## Data Availability

The data that support the findings of this study are available on request from the corresponding author. The data are not publicly available due to privacy or ethical restrictions.

## Declarations

### Permission to reproduce material from other sources

No material is being reproduced.
